# Single-center experience with perioperative antibiotic prophylaxis and surgical site infections in kidney transplant recipients

**DOI:** 10.1186/s12879-022-07182-z

**Published:** 2022-03-01

**Authors:** Agata Ostaszewska, Piotr Domagała, Michał Zawistowski, Edyta Karpeta, Michał Wszoła

**Affiliations:** 1 Department of General and Transplantation Surgery, Medical University of Warsaw, Nowogrodzka 59, 02-006 Warsaw, Poland; 2 Foundation of Research and Science Development, Warsaw, Poland

**Keywords:** Risk factors, Surgical site infection, Kidney transplantation, Perioperative antibiotic prophylaxis, Cefazolin

## Abstract

**Background:**

Infections in kidney transplant recipients are particularly challenging owing to the immunosuppressive treatment, usually long history of chronic illness, comorbidities and prior exposures to antibiotics. Among the most common complications early after surgery are surgical site infections. The aim of this study was to identify risk factors and evaluate epidemiological data regarding surgical site infections. Moreover, we were able to compare the current results with historical data from our institution when different perioperative antibiotic prophylaxis was practiced.

**Methods:**

We conducted a retrospective case–control study in a group of 254 deceased donor renal graft recipients transplanted in a single Central European institution. We evaluated epidemiological findings and resistance patterns of pathogens causing surgical site infections. We used multivariable logistic regression to determine risk factors for surgical site infections.

**Results:**

We revealed no differences in baseline characteristics between patients with and without surgical site infections. Ten surgical site infections (3.9%) were diagnosed (six superficial incisional, two deep incisional, and two organ/space). Eight species (19 strains) were identified, most of which were multi-drug resistant (63%). The most common was extended-spectrum β-lactamase producing *Klebsiella pneumoniae* (26%). We showed that statistically significant differences were present between reoperated and non-reoperated patients (adjusted odds ratio: 6.963, 95% confidence interval 1.523–31.842, *P* = .012).

**Conclusions:**

Reoperation is an individual risk factor for surgical site infection after kidney transplantation. According to our experience, cefazolin-based prophylaxis can be safe and is associated with relatively low prevalence of surgical site infections.

## Background

Surgical site infection is one of the most common nosocomial infections and a considerable cause of morbidity, prolonged hospitalization, and higher costs of medical care [1–3]. Solid organ transplant recipients are particularly susceptible to this complication mainly owing to their immunosuppressive treatment, usually long history of chronic illness, comorbidities and prior exposures to antibiotics.

Despite the progress in infection control practices and the development of perioperative antibiotic prophylaxis strategies, surgical site infections remain among the most challenging problems in the early period after kidney transplantation. Transplant teams may implement many preventive strategies at each stage of medical care to mitigate the risk of wound contamination, one of which is perioperative antibiotic prophylaxis [4, 5]. There is no single universal protocol and each transplant center should optimize their prophylaxis according to the local resistance patterns as well as the infection and colonization status of both the donor and recipient [6]. It is estimated that surgical site infections occur in 3% to 11% of renal graft recipients [4].

In this study, we aimed to retrospectively identify risk factors and epidemiological data associated with posttransplant surgical site infections. We also shared our single-center Central European country experience with a new perioperative antibiotic prophylaxis protocol that was modified after publishing our previous report [7].

## Methods

### Study design and eligibility criteria

As a continuation of our previous research, we maintained similar assumptions and research methods that we used before [7]. We conducted a retrospective case–control study in deceased donor renal graft recipients. We included adult patients transplanted or retransplanted between October 2015 and August 2018 in the Department of General and Transplantation Surgery at the Medical University of Warsaw, Poland. The exclusion criteria were: multi-organ transplantation, early (up to 7 days from the hospital admission) in-hospital death or graftectomy not related to surgical site infection, and the use of Bricker ileal conduit. The reporting of our study conforms to the STROBE statement [8].

### Setting and data collection

All study participants were observed for at least 1 year after transplantation or until their death or graft loss. Medical records were analyzed to collect the following data: donors’ and recipients’ demographic data (age, sex, body mass, and height), comorbidities, and renal function. We identified the cause of donors’ death, time spent at the intensive care unit, the need to use catecholamines, presence of any type of infection (urine, blood, intubation tube, and preservation fluid cultures were collected). We retrieved data about the method of organ storage, cold and warm ischemic times, and the length of transplant procedure. Information regarding the studied patients included also: the use, modality, and dialysis vintage, past medical and transplant history, number of HLA mismatches with the received organ, details about immunosuppressive treatment, posttransplant renal function, and complications (surgical site and/or urinary tract infections, surgical procedures, delayed graft function, primary non-function, and episodes of biopsy-proven acute rejection).

Surgical site infection was determined in line with the Centers for Disease Control and Prevention definition [9]. It was stratified into superficial incisional, deep incisional, and organ/space infection [10]. Delayed graft function was defined as the need for dialysis within the first posttransplant week [11]. Primary graft non-function was diagnosed when persistent lack of transplanted kidney function was observed and the patient was dialysis-dependent immediately after surgery. Biopsy-proven acute rejection was identified and classified in compliance with the Banff 2017 criteria [12].

### Analysis of microbiological results

Wound swabs were obtained when surgical site infection was suspected or when it was advised by a transplant nephrologist, including cases where graft preservation fluid cultures were positive. Isolates were identified and characterized using standard microbiological methods. Samples were cultured under aerobic and anaerobic conditions at 35–37 °C for 24–48 h with commercially available blood agar (Difco, Detroit, MI, USA) and MacConkey agar (bioMérieux, Marcy l'Etoile, France). Schaedler (bioMérieux) and liquid medium Schaedler (bioMerieux) were used for aerobic culture. Anaerobic strains were cultured in the anaerobic chamber. Sabouraud Agar (Difco) was used for the fungi culture. Biochemical characteristics of culture strains were evaluated using API and/or BioMerieux ID, according to manufacturer’s instructions. We investigated the antibiotic sensitivity of cultured strains using the ATB (bioMerieux) system according to recommendations of the Clinical and Laboratory Standards Institute (CLSI) [13] and the European Committee on Antimicrobial Susceptibility Testing (EUCAST) [14]. We also performed additional testing procedures for multi-drug resistant strains (classified in line with the 2019 CDC Antibiotic Resistance Threats Report) [15]. The strains’ antibiotic sensitivity analysis was carried out for microorganisms pathogenic to humans.

### Organ preservation prior to transplant

Renal grafts were recovered in hospitals outside our transplantation center. Immediately after organ recovery and cooling to 4 °C, most kidneys were preserved by simple hypothermic storage in the University of Wisconsin solution and transported in a thermally stable container. Hypothermic machine perfusion was utilized when the following factors were present: poor in situ perfusion, use of kidney from an extended criteria donor, considerable risk of acute kidney injury, and anticipated cold ischemic time exceeding 24 h. The decision to use machine perfusion was limited by the availability of a disposable organ cassette and machine preservation fluid.

### Perioperative antibiotic prophylaxis

Patients undergoing kidney transplantation received a standard perioperative antibiotic prophylaxis that was adapted in our center since October 2015. Each recipient was given a 2.0 g single-dose first generation cephalosporin (cefazolin) via an intravenous infusion 30 min before the first skin incision. Additionally, all patients received oral sulfamethoxazole–trimethoprim (480 mg daily starting on the 4th postoperative day) as a *Pneumocystis* pneumonia prophylaxis.

### Transplant procedure

Kidneys were transplanted using the standard approach. Graft vessels were anastomosed end-to-side to external iliac vessels either on the left or right side. Lich-Gregoir technique was used for the ureterovesical anastomosis.

### Immunosuppressive treatment

Standard immunosuppressive regimen consisted of calcineurin inhibitor (either tacrolimus or cyclosporine started at an oral dose of 0.2 mg/kg and 10 mg/kg, respectively), prednisolone (started simultaneously with the vascular anastomosis at intravenous dose of 250 mg or 500 mg, depending on the patient’s weight), and mycophenolate mofetil or sodium (1.0 g and 720 mg twice daily, respectively). Calcineurin inhibitors’ doses were halved on the second posttransplant day and the subsequent dosing was dependent on the regular blood concentration monitoring. Prednisone was continued at the dose of 125 mg every 12–24 h until the third postoperative day when its oral supplementation was started (at an initial dose of 20 mg/day that was gradually lowered to reach the dose of 10–15 mg/day on the 30th day after surgery). Patients with panel reactive antibody scores exceeding 20% and/or with at least four HLA mismatches were given an induction immunosuppression therapy with thymoglobuline or basiliximab.

### Outcomes and study groups

We divided all patients into two groups: “SSI” with recipients who developed surgical site infection and “non-SSI” including those without that complication. Cases were defined as all patients with surgical site infections identified within the study period. Cases consisted of all remaining renal graft recipients who did not have surgical site infection within that time. No matching was planned for this study. Our main goal was to identify potential risk and/or protective factors for surgical site infection.

### Statistical analysis

The assumption of normal distribution, evaluated with the Shapiro–Wilk test, was not fulfilled for any of the continuous variables. Thus their medians with ranges were reported. For categorical variables, frequencies and percentages were presented. Mann–Whitney U test was applied to compare continuous variables between the SSI and non-SSI groups. For categorical variables, differences between the groups were assessed with the Chi-square or Fisher’s exact tests, as appropriate. Multivariable logistic regression was used to identify the predictors of surgical site infections as well as to identify and adjust for potential confounding factors. Backward elimination with a *P* value criterion of .157 was used [16, 17]. Missing data were deleted pairwise. *P* < .05 was regarded as statistically significant. All analyses were performed in the R statistical environment, version 4.0.0 (R Core Team, 2020) [18].

Ethical approval was obtained from the Bioethics Committee at the Medical University of Warsaw (KB239/2013).

## Results

### Study participants

Between October 2015 and August 2018, 285 patients were transplanted in our unit. 31 of them were excluded due to multi-organ transplantation (8 patients), early in-hospital death or graftectomy not related to surgical site infection (4 cases), the use of Bricker ileal conduit (2 individuals), and other reasons (living donor transplants, loss to follow-up, and missing data). In result, 254 graft recipients were included in the final analysis.

The study population consisted of 87 women and 167 men. The median age of the participants was 50 (range, 18–78). They received renal grafts from deceased donors with the median age of 52 (range, 5–80). Five of them received their kidneys from a pediatric donor (age less than 18 years old). Only one patient was treated with cyclosporine while others received tacrolimus. The organs were stored in simple hypothermia in 219 cases and with the use of machine perfusion in 29 (in 6 individuals the storage modality was unknown or mixed). The donor- and recipient-related characteristics (demographics, comorbidities, past medical history, transplanted organ characteristics, renal function, and transplant procedure details with outcomes) are presented in Tables 1, 2, and 3.

**Table 1:** Baseline characteristics of deceased donors and renal grafts

Deceased donor factors	Entire cohort (n = 254)	non-SSI (n = 244)	SSI (n = 10)	*P* value^†^
Age	52.0 [5–80]	52.0 [5–80]	55.0 [20–68]	.434
Sex				
Female, n, %	85 (33.5%)	81 (33.2%)	4 (40.0%)	.736
Male, n, %	169 (66.5%)	163 (66.8%)	6 (60.0%)	
BMI, kg/m^2^	25.0 [17–40]	25.0 [17–40]	24.5 [22–37]	.952
ICU stay, days	3 [1–20]	3 [1–20]	5 [1–9]	.281
SCA				
Without SCA, n, %	195 (76.8%)	188 (77.0%)	7 (70.0%)	.702
With SCA, n, %	59 (23.2%)	56 (23.0%)	3 (30.0%)	
Vasopressors/inotropes at ICU				
Catecholamines not used, n, %	21 (8.3%)	20 (8.2%)	1 (10.0%)	.585
Catecholamines used, n, %	233 (91.7%)	224 (91.8%)	9 (90.0%)	
Hypertension				
Without HTN, n, %	164 (65.1%)	160 (66.1%)	4 (40.0%)	.102
With HTN, n, %	88 (34.9%)	82 (33.9%)	6 (60.0%)	
Creatinine level, mg/dl	1.00 [0.41–4.80]	1.00 [0.41–4.8]	1.10 [0.62–3.49]	.376
ECD				
Standard donor, n, %	173 (68.1%)	168 (68.9%)	5 (50.0%)	.297
ECD, n, %	81 (31.9%)	76 (31.1%)	5 (50.0%)	
Perfusion type				
Simple hypothermia, n, %	219 (88.3%)	210 (88.2%)	9 (90.0%)	> .999
CPPH, n, %	29 (11.7%)	28 (11.8%)	1 (10.0%)	
CIT, min	1042 [285–2350]	1020 [285–2350]	1176 [840–1730]	.275
WIT, min	35 [18–75]	35 [18–75]	33 [22–45]	.620

*BMI* body mass index, *ICU* intensive care unit, *SCA* sudden cardiac arrest, *HTN* arterial hypertension, *ECD* extended criteria donor, *CPPH* continuous pulsatile perfusion in hypothermia, *CIT* cold ischemic time, *WIT* warm ischemic time

^†^Two-sided *P* values are reported. The Fisher’s exact test was used for the categorical variables and Mann–Whitney U for the continuous ones

**Table 2:** Baseline characteristics of kidney transplant recipients

Recipient factors	Entire cohort (n = 254)	Non-SSI (n = 244)	SSI (n = 10)	*P* value^†^
Age	50.0 [18–78]	49.5 [18–78]	57.0 [28–70]	.427
Sex				
Female, n, %	87 (34.3%)	84 (34.4%)	3 (30.0%)	> .999
Male, n, %	167 (65.7%)	160 (65.6%)	7 (70.0%)	
Body Mass Index, kg/m^2^	25.0 [17.0–34.0]	25.0 [17.0–34.0]	28.0 [20.0–33.0]	.096
Dialysis vintage, months	24.5 [0–166]	24.0 [0–166]	32.5 [0–51]	.813
Dialysis method				
Peritoneal, n, %	16 (6.3%)	15 (6.1%)	1 (10.0%)	.840
Hemodialysis, n, %	202 (79.5%)	195 (79.9%)	7 (70.0%)	
Combined, n, %	13 (5.1%)	12 (4.9%)	1 (10.0%)	
Pre-emptive KTx, n, %	22 (9.1%)	22 (9.0%)	1 (10.0%)	
First KTx or retransplantation				
First KTx, n, %	220 (87.0%)	211 (86.8%)	9 (90.0%)	> .999
Retransplantation, n, %	33 (13.0%)	32 (13.2%)	1 (10.0%)	
HLA mismatch				
HLA ≤ 3, n, %	167 (65.7%)	161 (66.0%)	6 (60.0%)	.739
HLA > 3, n, %	87 (34.3%)	83 (34.0%)	4 (40.0%)	
Induction therapy				
No induction, n, %	160 (82.5%)	151 (81.6%)	9 (100%)	.364
With induction, n, %	34 (17.5%)	34 (18.4%)	0 (0.0%)	
Hypertension				
Without HTN, n, %	19 (7.5%)	19 (7.8%)	0 (0.0%)	> .999
With HTN, n, %	234 (92.5%)	224 (92.2%)	0 (0.0%)	
Ischemic heart disease				
Without CAD, n, %	199 (78.7%)	190 (78.2%)	9 (90.0%)	.694
With CAD, n, %	54 (21.3%)	53 (21.8%)	1 (10.0%)	
HBV				
HBV negative, n, %	219 (89.4%)	213 (89.9%)	6 (75.0%)	.204
HBV positive, n, %	26 (10.6%)	24 (10.1%)	2 (25.0%)	
HCV				
HCV negative, n, %	249 (98.0%)	239 (98.0%)	10 (100%)	.817
HCV positive, n, %	5 (2.0%)	5 (2.0%)	0 (0.0%)	

^†^Two-sided *P* values are reported. The Fisher’s exact test was used for the categorical variables, except for the dialysis method where Chi-square test was utilized, and Mann–Whitney U for the continuous ones

**Table 3:** Transplantation outcomes

Post-transplant factor	Entire cohort (n = 254)	Non-SSI (n = 244)	SSI (n = 10)	*P* value^†^
DGF				
Without DGF, n, %	138 (54.5%)	133 (54.7%)	5 (50.0%)	> .999
With DGF, n, %	115 (45.5%)	110 (45.3%)	5 (50.0%)	
PNF				
Without PNF, n, %	252 (99.6%)	242 (99.6%)	10 (100%)	> .999
With PNF, n, %	1 (0.4%)	1 (0.4%)	0 (0.0%)	
Lymphocele				
No lymphocele, n, %	192 (96.5%)	184 (96.8%)	8 (88.9%)	.280
With lymphocele, n, %	7 (3.5%)	6 (3.2%)	1 (11.1%)	
Hematoma				
Without hematoma, n, %	145 (73.2%)	152 (75.1%)	3 (33.3%)	.012
With hematoma, n, %	53 (26.8%)	47 (24.9%)	6 (66.7%)	
Need for reoperation at early follow-up				
Reoperated, n, %	29 (11.4%)	24 (9.8%)	5 (50.0%)	.002
Non-reoperated, n, %	225 (88.6%)	220 (90.2%)	5 (50.0%)	
UTI				
Without UTI, n, %	156 (61.4%)	152 (52.3%)	4 (40.0%)	.139
With UTI, n, %	98 (38.6%)	92 (37.7%)	6 (60.0%)	
Serum creatinine on the 30th POD	1.64 [0.51–8.49]	1.63 [0.51–8.49]	2.06 [0.89–5.44]	.319
Serum creatinine on the 90th POD	1.49 [0.64–8.30]	1.49 [0.64–8.30]	1.77 [1.01–7.99]	.162
Serum creatinine on the 180th POD	1.48 [0.67–7.59]	1.47 [0.67–7.59]	1.76 [0.99–3.34]	.445
Serum creatinine on the 360th POD	1.46 [0.71–6.00]	1.44 [0.71–6.00]	1.70 [0.96–3.26]	.355
Kidney function 1 year after transplantation				
Graft survival less than 1 year, n, %	10 (4.9%)	8 (4.1%)	2 (22.2%)	.064
Graft survived 1 year, n, %	196 (95.1%)	189 (95.9%)	7 (77.8%)	
MDRD on the 30th POD	43.1 [7.34–142.00]	43.1 [7.34–142.00]	36.8 [8.73–106.00]	.387
MDRD on the 90th POD	48.8 [6.89–109]	49.0 [6.89–109.00]	41.8 [7.14–80.60]	.230
MDRD on the 180th POD	49.9 [8.17–122.00]	50.0 [8.17–122.00]	48.2 [23.50–78.80]	.684
MDRD on the 360th POD	51.3 [7.92–141.00]	51.5 [7.92–141.00]	48.3 [24.20–85.50]	.481
Time of hospital stay, days	7 [0–42]	7 [0–38]	7 [4–42]	.347

*DGF* delayed graft function, *PNF* primary graft non-function, *UTI* urinary tract infection, *MDRD* MDRD eGFR, *POD* postoperative day

^†^Two-sided *P* values are reported. The Fisher’s exact test was used for the categorical variables and Mann–Whitney U for the continuous ones

Ten patients were diagnosed with surgical site infection and thus included in the SSI group. The non-SSI group consisted of 244 patients. The baseline characteristics between the two cohorts were compared and no statistically significant differences were noted apart from the size of each group (Tables 1, 2, and 3). Having met the predefined study requirements, we proceeded to the further analysis.

### Primary outcome

Clinically- and laboratory-confirmed surgical site infections were observed in 10 patients (10/254, 3.9%). Six patients were diagnosed with superficial incisional (6/10, 60%), two with deep incisional (2/10, 20%), and two with organ/space infection (2/10, 20%). What we observed is that surgical site infections occurred mainly in patients with complications that often required reoperation before being diagnosed with surgical site infection (*P* = .002). Four patients were diagnosed with primary surgical site infection, three had an urinary complication, in two the pathogens were transferred with the transplanted organ, and one had an iatrogenic complication (colon perforation during the initial skin incision). We summarized the findings in those patients in Table 4.

**Table 4:** Patients diagnosed with surgical site infection

Case no	Sex	Age (range 28–70)	Suspected source of SSI	Identified bacterial species	Postoperative course	Outcome
1	M	> 60	Iatrogenic (colon perforation)	*Klebsiella pneumoniae* *Enterococcus faecium* *Bacteroides vulgatus*	Laparotomy on POD 12, caecum resection with ileostomy, V and A therapy	PNF, graftectomy on POD 21
2	M	> 60	Bacteria donor transfer	*Acinetobacter baumannii*	Eventration, V and A therapy	Recovery
3	M	30–60	Bacteria donor transfer	*Acinetobacter baumannii*	Subcutaneous abscess, V and A therapy	Recovery
4	F	30–60	Urinary complication	*Acinetobacter baumannii*	Graft revision on POD 20—urinary fistula repair, 2nd graft revision on POD 33—ureteral necrosis, V and A therapy	Graftectomy on POD 33
5	M	< 30	Urinary complication	*Klebsiella pneumoniae*	Graft revision on POD 19—urinary fistula repair, A therapy	Recovery
6	F	> 60	Urinary complication	*Escherichia coli*	Graft revision on POD 11—urinary fistula repair, V and A therapy	Recovery
7	M	30–60	Primary SSI	*Klebsiella pneumoniae* *Enterobacter cloacae*	A therapy	Recovery
8	F	> 60	Primary SSI	*Escherichia coli* *Enterobacter cloacae* *Enterococcus faecalis* *Finegoldia magna*	A therapy	Recovery
9	M	< 30	Primary SSI	*Klebsiella pneumoniae* *Enterococcus faecalis*	A therapy	Recovery
10	M	< 30	Primary SSI	*Klebsiella pneumoniae* *Enterococcus faecium* *Escherichia coli*	V and A therapy	Recovery

*SSI* surgical site infection, *M* male, *F* female, *POD* postoperative day, *PN*, primary graft non-function, *KTx* kidney transplantation, *V* vacuum, *A* antibiotic

Superficial incisional infections were treated with intravenous antibiotics, surgical debridement, and secondary wound closure was performed (except in one case when vacuum therapy with subsequent secondary wound sewing was implemented). Initially empirical antibiotic treatment was adjusted depending on the microbiological findings.

The two recipients with deep incisional infections were treated similarly but both underwent the vacuum therapy. One patient developed fascial disruption that was managed surgically.

Organ/space infections were all successfully treated with adequate antibiotics (carbapenem with vancomycin; linezolid was used only once), surgical (including fascial repair), and vacuum therapies. We identified major complications that are suspected to cause the infection in the two individuals: iatrogenic caecal perforation in one case and urinary anastomosis complication in the second (Table 4).

### Surgical site infections—etiopathology

In Table 5, we present the list of pathogens detected by wound swabs collected from the SSI patients. Microbiological analyses revealed the presence of eight bacterial species (19 strains). The most common was extended-spectrum β-lactamase (ESBL) producing *Klebsiella pneumoniae* (five isolates, 26%). Ten out of 14 (71%) Gram negative pathogens were ESBL positive. 12 strains (12/19, 63%) were multi-drug resistant [15]. In two recipients surgical site infections were caused by bacteria transferred with the grafts (the same pathogen, *Acinetobacter baumannii*, was detected in the preservation fluid and wound swab specimens). One of them developed deep incision infection and required reoperation followed by vacuum therapy and antibiotics. The second had superficial infection cured successfully with conservative antibiotic treatment.

**Table 5:** Pathogens identified by wound swabs in the SSI group

Gram’s stain	Bacterial species	Resistance phenotypes	Frequency	Type of surgical site infection		
				Superficial	Deep	Organ/space
Positive (5/19)	*Enterococcus faecalis*		2	1		1
	*Enterococcus faecium*	HLAR	2	1		1
	*Finegoldia magna*		1	1		
Negative (14/19)						
	*Escherichia coli*		3	2	1	
	*Enterobacter cloacae* compl	ESBL+	2	2		
	*Klebsiella pneumoniae*	ESBL+	5	4		1
	*Bacteroides vulgatus*		1			1
	*Acinetobacter baumannii* compl	ESBL+	3	1	1	1
			19	12	2	5

*HLAR* high-level aminoglycoside resistance, *ESBL* extended-spectrum beta-lactamase

### Transplant outcomes

There was no difference in the occurrence of delayed graft function, primary non-function, and creatinine serum levels between the analyzed groups after transplantation (Table 3). Appearance of hematoma in the operated area strongly correlated with the occurrence of surgical site infection (*P* = .012). Moreover, surgical site infections were more frequent if reoperation in the early posttransplant period was performed (*P* = .002).

### Risk factors for surgical site infection

We performed a multivariable logistic regression analysis to identify the risk factors for surgical site infections (Table 6). It resulted in the inclusion of two exposures in the model: reoperation and presence of hematoma in the operated area. Although the two variables are correlated, χ^2^ (1, n = 198) = 13.217, *P* < .001, both could be included owing to the low value of variance inflation factor (VIF = 1.075). Additionally, BMI was included in that model as a known confounding factor. Therefore, we showed that there are statistically significant differences between reoperated and non-reoperated patients (adjusted odds ratio: 6.963, 95% confidence interval 1.523–31.842, *P* = .012).

**Table 6:** Logistic regression model for surgical site infection as a dependent variable

Predictor (role)	Estimate	SE	Z	*P* value	OR	95% CI	
						Lower	Upper
Intercept	− 9.547	2.913	− 3.277	.001	7.143e−5	2.366e−7	0.022
Reoperation (exposure)	1.941	0.776	2.502	.012	6.963	1.523	31.842
Hematoma in the surgical site (exposure)	1.365	0.787	1.733	.083	3.916	0.837	18.329
BMI (confounder)	0.205	0.104	1.966	.049	1.228	1.001	1.506
R^2^_N_: 0.263; AIC: 64.43; Accuracy: 96.0%; AUC: 0.857; Overall Model Test, χ^2^: 16.79, df: 3, P < .001							

*SE* standard error, *OR* odds ratio, *CI* confidence interval, *BMI* body mass index, *R*^*2*^_*N*_ Nagelkerke’s R^2^, *AIC* Akaike information criterion, *AUC* area under the curve

## Discussion

In this study, we summarized our experience with surgical site infections and perioperative antibiotic prophylaxis consisting of the first generation cephalosporin administered at a single dose 30 min prior to the initial skin incision. In the studied group of 254 deceased donor renal graft recipients, 10 (3.9%) developed surgical site infection. We calculated that reoperation adjusted for the presence of hematoma in the surgical site and BMI is a risk factor for this challenging complication (adjusted odds ratio: 6.963, 95% confidence interval 1.523–31.842, *P* = .012).

Surgical site infections occur in 3% to 11% of patients after kidney transplantation [3–5, 9, 19]. The prevalence of 3.9% in our transplant unit seems, therefore, to be relatively low. Even though the complication rarely leads to graft loss, it is proved to significantly increase morbidity and prolonged hospitalization time [20].

The identification of certain surgical site infection risk factors has been a significant problem for years as the conclusions vary in different publications. Most of them mention body mass index (BMI), immunosuppression regimen, older age, diabetes and accompanying heart diseases, cold ischemic time, duration of surgical procedure, and delayed graft function [20–23]. In this study, we aimed to verify those findings and identify potentially new predicting factors based on our experience.

While evaluating the impact of BMI on surgical site infections, we expected that patients with a higher BMI would acquire more episodes of surgical site infections. Obese patients are proved to have an increased prevalence of postoperative wound colonization, as demonstrated by multiple studies [24–27]. Some data on intraabdominal surgery show that especially the thicker subcutaneous fat tissue is related with higher wound morbidity [28]. In our study, we observed that the median BMI was higher in patients with surgical site infections compared to those without infection but the difference was not statistically significant (28.0 vs 25.0, U = 842.0, *P* = .096). However, BMI was indeed confirmed to be an important confounding factor that was included in our final logistic regression model.

Literature clearly confirms that patients with more intense immunosuppression are expected to acquire more surgical site infections [25]. In current study, each patient received the same, triple-drug immunosuppressive treatment, and almost 88% (224/254) of them did not receive antibody induction. As the protocol was universal, no differences could be found between the studied groups and we could not identify more intensive immunosuppression to be a predisposing risk factor for SSI. Similar conclusions can be drawn regarding patients’ age. However, reoperation was confirmed as a major risk factor for surgical site infection [25, 29]. It is most likely caused due to increased exposure of the surgical site on contamination [20]. In such circumstances, the immunocompromising influence of anti-rejection drugs worsens the outcomes [29]. We detected that the presence of a hematoma in the operated area also predisposes to the development of infection. This is not a frequent observation in the literature. In fact, the two variables (hematoma and reoperation) are correlated (not strongly, VIF = 1.075) as often they can cause one another. The multivariable logistic regression analysis confirmed that reoperation is an independent risk factor for surgical site infection. Therefore, we provided an odds ratio adjusted for the occurrence of hematoma in the surgical site and patient’s BMI. That leads us to the point, where in the future, this observation may be helpful in postoperative estimation of the risk of infection wound problems.

Evidence of the beneficial role of perioperative antibiotic prophylaxis in reducing postoperative surgical site infections in solid organ recipients is well-established [5, 7, 19]. Only a few reports question its efficacy [9, 30]. However, specific antibiotic regimens and durations vary widely across transplant centers and procedures, and the quality of the evidence supporting specific practices is quite poor [5, 31–33]. Currently, there are no strict formal recommendations on perioperative antibiotic prophylaxis administration in kidney recipients. According to the IDSA/ASHP/SIS/SHEA guidelines, each transplant center should customize their own protocol that corresponds with their unique microbiological setting (patients’ and donor’s colonization statuses, local resistance patterns, and presence of nosocomial pathogens) [5]. It should also be pointed out that such prophylaxis can lead to some problems including increased: financial costs, rates of *Clostridioides difficile* colonization and colitis, incidence of antimicrobial resistance, and other adverse events [34–37]. Thus, targeted antibiotics should be used in each case based on individual risk factors and local patterns of resistance [38–40]. Moreover, the spectrum of organisms involved in SSI in kidney recipients is more diverse than for the general population due to other important factors such as the underlying end‐stage organ failure, immunosuppression, prolonged hospitalization, organ transportation/preservation, and previous exposures to antibiotics in donors and recipients that could predispose to infections with multi-drug resistant organisms [15]. Therefore, the use of antibiotic prophylaxis should be optimized as to be truly effective yet still minimal as to restrain the spread of multi-drug resistant pathogens. As we modified our local protocol a few years earlier, we had a unique chance to compare our historical data with the current ones.

Before October 2015 our strategy was to administer 2.0 g of the third generation cephalosporin (ceftriaxone) in a single infusion 30 min prior to the first skin incision. We observed back then that surgical site infections occurred in 12 of 214 patients (5.6%) [7]. The historic incidence is slightly higher, although we think that the difference is not clinically significant. This finding is very satisfying as we started using an antibiotic with a narrower spectrum and achieved similar results. We hope that such an approach can at least minimally help to decrease the risk of development of new multi-drug resistant strains. Similar conclusions were demonstrated by other authors [4, 21]. Furthermore, other factors like: shortening surgical operative time, optimal sterile technique, proper perioperative management of patients’ comorbidities as well as good glucose and temperature regulation, are also assumed to be imperative to limit surgical site infections but those elements were outside the scope of our research [41–45].

### Limitations

The main limitations of our study are the limited number of cases and the retrospective, non-randomized design carried out at a single Central European institution. The incompleteness of medical records resulted in the presence of some missing data. Therefore, we cannot exclude bias due to missing data. Another bias that might be present in such case–control study is caused by confounding [46]. We attempted to eliminate it by restriction (e.g., we excluded patients with Bricker ileal conduit due to significant deviation from a standard kidney transplant procedure that carries a higher risk for surgical site infection) and multivariable logistic regression (i.e., we calculated adjusted odds ratio).

Another limitation is related to the lack of definition of minimal clinically important difference (MCID) regarding the comparison of different perioperative antibiotic prophylaxis regimens. Such a concept would help transplant centers to confirm when a change in their perioperative antibiotic prophylaxis is reasonable. We were able to compare our current prophylaxis policy to the previous one concerning the prevalence of surgical site infection, although it was not the main scope of our study. We conclude that the decrease in prevalence of surgical site infection of 1.7% is not clinically relevant [47]. The difference is also not statistically significant (data not shown) which increases our confidence in that matter. However, if MCIDs were defined by transplant societies regarding the outcomes of perioperative antibiotic prophylaxis, it could possibly improve the process of choosing the most optimal regimen in each transplant center [48].

### Conclusions

In conclusion, our study identifies reoperation as a strong risk factor for surgical site infection. Hematoma in the operated area also correlates with higher incidents of this complication. Our positive experience with single-dose cefazolin-based perioperative antibiotic prophylaxis shows that it can be a safe and effective method that in our unit is associated with relatively low prevalence of SSI (3.9%).

## Data Availability

The datasets used and/or analyzed during the current study are available from the corresponding author on reasonable request.

## Abbreviations

BMI: Body mass index
ESBL: Extended-spectrum β-lactamase
HLA: Human leukocyte antigen
SSI: Surgical site infection
VIF: Variance inflation factor
